# Psycho-Physiological Effects of a Peony-Viewing Program on Middle-Aged and Elderly Individuals at Different Phenological Stages

**DOI:** 10.3390/ijerph16030439

**Published:** 2019-02-02

**Authors:** Ren-Lin Zhao, Gang Zhang, Xi Wang, Bo-Tong Zhang, Li-Na Guo, Li-Xin Niu, Yan-Long Zhang

**Affiliations:** College of Landscape Architecture and Arts, Northwest A&F University, Yangling 712100, China; zhaorenlin@nwafu.edu.cn (R.-L.Z.); zhan11a@163.com (G.Z.); wangxi9311@163.com (X.W.); bonizzhang@163.com (B.-T.Z.); guolina0811@163.com (L.-N.G.)

**Keywords:** tree peonies, phenological period, campus environment, biofeedback therapy, Profile of Mood States, State–Trait Anxiety Inventory, middle-aged and elderly

## Abstract

To address the question of whether the behavior of humans to view different phenological peony flowers has various effects on their physical and mental parameters, we investigated psycho-physiological responses of 74 participants (61.3 ± 10.78 years old) to environments of pre- and post-viewing tree peonies at four stages, including the exhibition leaf stage (ELS), initial bloom stage (IBS), full bloom stage (FBS), and terminal bloom stage (TBS). Physiological factors were examined using systolic blood pressure (SBP), diastolic blood pressure (DBP), heart rate (HR), fingertip pulse (FP), blood oxygen saturation (SpO_2_), and psychological evaluation, which was carried out using the Profile of Mood States (POMS) and State–Trait Anxiety Inventory (STAI). The results indicated that the SBP, DBP, HR, and FP levels of participants were significantly reduced after viewing tree peonies, whereas no remarkable alterations in SpO_2_ were found. The POMS scores of anger–hostility (A–H), fatigue–inertia (F–I), tension–anxiety (T–A), confusion–bewilderment (C–B), and depression–dejection (D–D) were significantly lower, but of vigor–activity (V–A) was higher post-viewing than pre-viewing. Furthermore, participants exhibited markedly decreased anxiety levels according to the STAI. Notably, the changes in measurement indices were more pronounced at the FBS. Our studies demonstrated that a short peony-viewing program, especially at the FBS with completely opened and large tree peony flowers, would be a promising therapeutic method for improving physiological functions as well as an effective psychological relaxation strategy for middle-aged and elderly individuals.

## 1. Introduction 

Human beings have been inextricably linked to the natural environment [1]. A growing body of scientific evidence indicates that direct or indirect interaction with nature or plants is beneficial to human health and well-being [2,3,4,5]. Simultaneously, environments with natural elements can better relieve stress and mental fatigue than those without natural elements [6]. Since the 1970s, horticultural therapy has been developed rapidly in Europe, America, Japan, and other countries, mainly relying on medical instruments and psychological methods to study the effects of plants on human psycho-physiological health through two aspects of human sensory stimulation (vision, olfaction, touch, audition, and taste) and horticultural practice [7,8]. These studies have shown that plants and gardening have significant therapeutic effects on physical and mental health. 

The natural environment produces a positive parasympathetic nervous system reaction that is related to physiological function recovery [9,10]. Several studies have suggested that exposure to nature or plants leads to physiological improvement. Participants in a forest environment exhibit lower pulse rates, diastolic blood pressure (DBP), and systolic blood pressure (SBP) than those in urban settings [11,12,13,14,15,16,17,18]. Subjects viewing natural scenery videos have lower heart rates (HRs) and blood pressure than those watching urban landscape videos [2]. Patients who lived in rooms with plants, compared to rooms without plants, display reduced SBP and HRs [6,19,20,21]. There is considerable evidence that contact with nature or plants has psychological benefits for relieving stress and mental fatigue, improving positive mood (such as happiness and calmness), reducing negative emotion (such as fear, sadness, anger, intensity, and anxiety) [22,23,24,25,26,27,28], and enhancing environmental satisfaction [29]. 

The positive effects of nature and plants on people’s physiology and psychology have been well documented, as described above. However, limited studies have been performed to examine the effects of colors and volatiles of plants on the people who view and smell them. Recently, the visual effect of plant color has been explored. Different plantscape colors are found to stimulate different psycho-physiological reactions, including making a place more pleasant, more exciting, and brighter [30,31], as well as reducing SBP and DBP, and promoting the level of vigor [32]. Participants who viewed flowers recover from stress faster [33] and tend to experience a calming and relaxing state of mind [34]. In addition, the stimulation of essential oils or plant scent inhalation affects human physiology and psychology, which mainly includes putting people in a relaxed state, improving their work efficiency, and bringing a person’s body and mind into a balanced and harmonious condition [35,36,37,38]. Therefore, understanding the effects of pants (colors and volatiles) on human psycho-physiological responses is imperative. Furthermore, most studies have been accomplished in the laboratory [30,31,32] and carried out at a period of plant growth [34]. Evidence-based research clarifies that the psycho-physiological effects of plants in different growth periods on humans are limited [39,40]. However, with the annual growth cycle, the colors, volatiles, and morphology of plants at the flowering stages are changing constantly and might impose an influence on psycho-physiological responses, which seems to be the gap in the research. 

The tree peony is an important ornamental plant with colorful flowers and rich fragrances. Peony-viewing programs have a long history in China. Many people enjoy appreciating tree peonies at the flowering stage and expressing their feelings through painting and versifying [41]. Why are peony-viewing programs so charming? What is the relationship between peony-viewing and human physical and mental health? A previous study has examined the effect of a peony-viewing program on students at the full bloom stage (FBS) [42]. The present study therefore investigated middle-aged and elderly individuals’ physiological and psychological responses to peony-viewing programs at different phenological stages (exhibition leaf stage (ELS), initial bloom stage (IBS), full bloom stage (FBS), and terminal bloom stage (TBS)). Physiological indices and mood states were measured before and after the short peony-viewing program in a peony garden provided by the Northwest A&F University, P. R. China. Our focus was on a special type of peony garden that is not primitive or wild in nature but is a semi-managed or fully managed green space on campus. This study sought to understand the effects of the peony-viewing program on middle-aged and elderly individuals, and to provide a theoretical basis for the healthy campus life environment construction.

## 2. Materials and Methods

### 2.1. Participants 

This study was performed with 74 middle-aged and elderly subjects aged 44 to 83 years (61.3 ± 10.78; mean ± SD), consisting mainly of faculty members and senior citizens with normal visual acuity and no history of neurological illness. None of the subjects had abnormalities affecting smell (e.g., pollen allergy, stronger/weaker senses of smell), and they were asked not to use any sprays or perfumes prior to the experiments [43]. The gender distribution of participants was 37 females (50%) and 37 males (50%). The participants’ personal information and characteristics are listed in Table 1. A questionnaire [34] used to evaluate participants’ health conditions before the test included the following questions: “Before the test, did you stay up late?”, “Eight hours before the test, did you drink alcohol?”, “Eight hours before the test, did you take any (antihypertensive) drugs?”, and “eight hours before the test, did you have coffee or similar beverages?”. These health factors may influence physiological testing and were used for exclusion criteria before the experiments. The participants answered all the questions during every experiment of each phenological stage of peony. In addition, the study was conducted in accordance with the ethics rules of Northwest A&F University.

### 2.2. Experimental Setting

**Figure 1:**
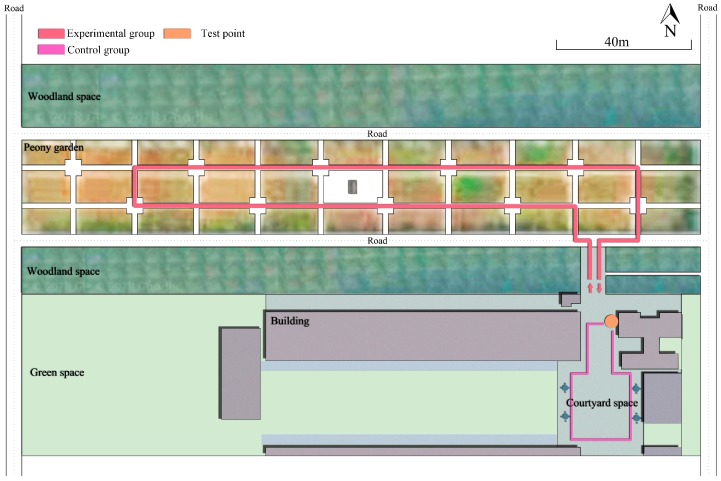
The environmental settings of the control and experimental groups on a location map.

**Figure 2:**
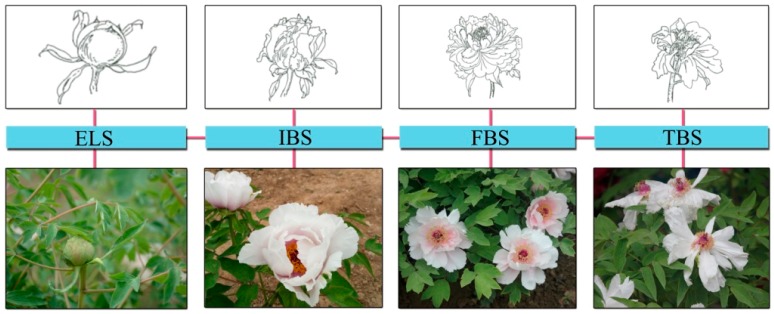
Morphological characteristics of peony flowers at the different stages. ELS: exhibition leaf stage, IBS: initial bloom stage, FBS: full bloom stage, and TBS: terminal bloom stage.

The research was conducted in the peony garden (Figure 1), which was surrounded by four roads. The peony garden had roads, seats, a rest square, and a number of tree peony cultivars. It was 215 m long (east–west) by 30 m wide (north–south) and covered an area of approximately 0.67 ha. The tree peonies were approximately 8–10 years old [44]. The height of the trees was 0.5 ± 0.1 m, and the number of blooms per tree was 18 ± 6. This study was conducted separately at the exhibition leaf stage (ELS), initial bloom stage (IBS), full bloom stage (FBS), and terminal bloom stage (TBS). The leaf buds of the whole tree were basically expanded at the exhibition leaf stage. Most peony petals were slightly opened, but a few had already opened and were at the initial bloom stage. The peony petals were all opened to the maximum extent at the full bloom stage. Most of the peony petals began to fade at the terminal bloom stage [44]. The morphological characteristics of peony flowers were shown in Figure 2. During the peony-viewing program, there was no rain and the weather was pleasant with a temperature of 22.7 ± 1.6 °C, a relative humidity of 54.3 ± 2.5%, and a wind speed of 1.5 ± 0.3 m/s. In addition, the roads around the peony garden were closed for traffic by the campus managers to keep a good environmental condition for the experiments. 

### 2.3. Research Design 

We adopted a one-group pretest–posttest field experimental design to evaluate the participants’ physiological activity and mood states incurred by viewing tree peonies. Specifically, all participants’ physiological and psychological responses were measured before they viewed tree peonies (baseline), and post-program measures were obtained upon their return. The effects of viewing tree peonies on physiological activity and mood states could therefore be identified by comparing the baseline and posttest measurements.

**Figure 3:**
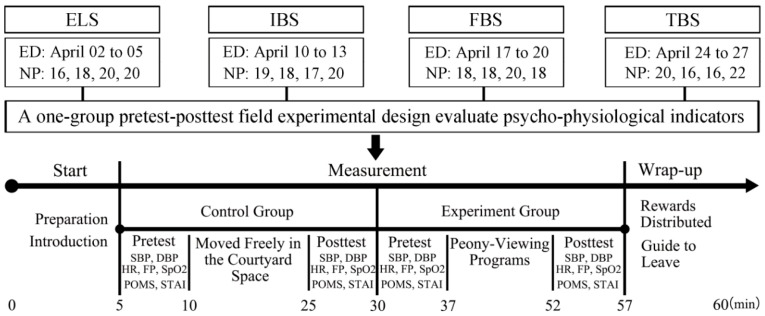
Sequence and contents of associated experimental events. ED: experimental date, NP: number of participants, ELS: exhibition leaf stage, IBS: initial bloom stage, FBS: full bloom stage, and TBS: terminal bloom stage. All subjects were examined individually.

The experiments were conducted when the tree peonies were at the exhibition leaf stage (ELS), initial bloom stage (IBS), full bloom stage (FBS), and terminal bloom stage (TBS), severally, from 9:00 am to 12:00 pm on 2–27 April 2018. Four tests were carried out at each phenological stage, respectively (Figure 3). The participants gathered at a designated location. Before the start of the experiment, the participants were allowed to move freely in the courtyard space (with hard pavement, a stone table, and several buildings, but without peonies (Figure 1)) for 15 min [16]. Their pre- and post-psycho-physiological indicators were recorded, and they were treated as a control group. Then, all participants took part in the experiment in a prearranged order and entered the peony garden to watch tree peonies. Their pre- and post-viewing physiological and psychological indicators were recorded. A guided 15-min peony-viewing program was organized to include the major stimulation of two senses, namely, vision (e.g., peony leaves, flower colors) and olfaction (e.g., the volatiles of peonies). During this process, subjects were allowed to touch the tree peonies, but not to talk with others, look at the phone, and walk out of the peony garden. Each participant walked for approximately 300 m in the peony garden. The specific steps are as follows (Figure 2). 

### 2.4. Physiological Indices

A biofeedback measurement method was used as previously described [28], based on a variety of advanced electronic instruments to display the body’s internal physiological activities stimulated by the visual or auditory signals. This method has been widely used in the field of people–plant relationships to examine the changes of physiological conditions of subjects. In our study, participants’ systolic blood pressure (SBP), diastolic blood pressure (DBP), and heart rate (HR) were measured by sphygmomanometer (Omron, HEM-7136, Kyoto, Japan) [42]. A fingertip pulse oximeter (M70C) was used to record the fingertip pulse (FP) [32] and blood oxygen saturation (SpO_2_) [45]. 

Blood pressure and HR are the most commonly used indicators to detect the health status of the cardiovascular system, reflecting the activity of the human autonomic nervous system including the sympathetic nervous system (SNS) and the parasympathetic nervous system (PNS) [32]. In general, the SNS is associated with accelerated HR and increased blood pressure, and the PNS is associated with slower HR and lower blood pressure. An increased HR in humans represents an increase in the degree of tension and physiological arousal.

### 2.5. Psychological Indices

A psychological test method [46] was employed to observe the psychological and social phenomena of groups or individuals using a specific psychological rating scale and to quantitatively evaluate and explain the results. In this experiment, two psychological scales, the State–Trait Anxiety Inventory (STAI) [28] and the Profile of Mood States (POMS) [37], were used to test subjects’ psychological conditions. The STAI questionnaire was composed of 20 questions, each with a score of 1–4 points. Participants’ state–anxiety scores were determined by summing up their ratings of the 20 questions (e.g., I feel comfortable; I feel confused; I am satisfied). Higher scores indicated higher anxiety levels. The POMS questionnaire consisted of 35 questions and was divided into 6 dimensions: anger–hostility (A–H), depression–dejection (D–D), tension–anxiety (T–A), confusion–bewilderment (C–B), vigor–activity (V–A), and fatigue–inertia (F–I). A higher score for each dimension indicated a higher degree of the specified emotion.

### 2.6. Data Analysis

Data were analyzed by SPSS 19.0 (IBM^®^ SPSS^®^ Statistics, Armonk, NY, USA). A paired sample *t*-test was used to compare the means for psycho-physiological data in all experimental groups and control groups. The psycho-physiological changes between the experimental groups at different stages were determined by one-way analysis of variance (ANOVA). Partial eta squared (*η_p_*^2^) was used to report the estimate of effect sized for the ANOVA. The data were expressed as the mean ± standard deviation (mean ± SD). In all comparisons, a *p*-value of <0.05 was considered statistically significant. Effect size was reported using Cohen’s *d*. 

## 3. Results

**Figure 4:**
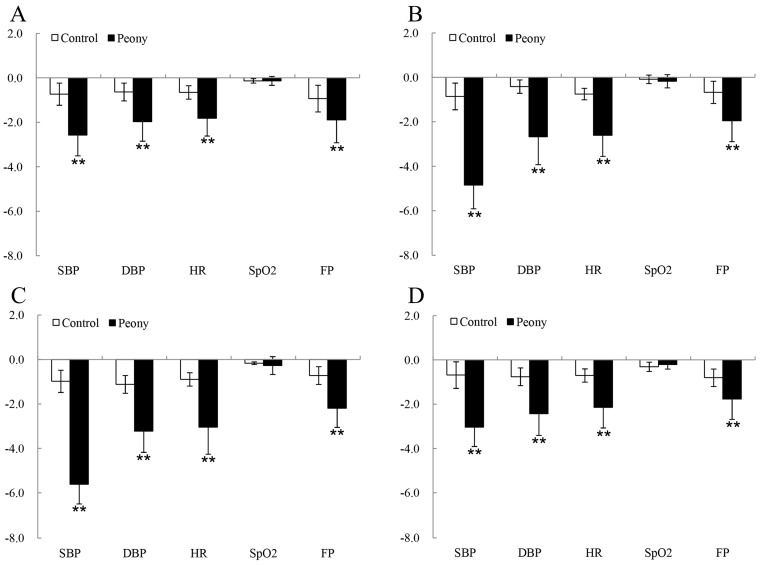
The pre- and post-viewing changes of participants’ physiological indices in the experimental (peony) and control groups at (**A**) ELS (exhibition leaf stage), (**B**) IBS (initial bloom stage), (**C**) FBS (full bloom stage), and (**D**) TBS (terminal bloom stage). SBP: systolic blood pressure, DBP: diastolic blood pressure, HR: heart rate, SpO_2_: blood oxygen saturation, and FP: fingertip pulse. N = 74, mean ± SD. ** *p* < 0.01, paired-sample *t*-test.

**Figure 5:**
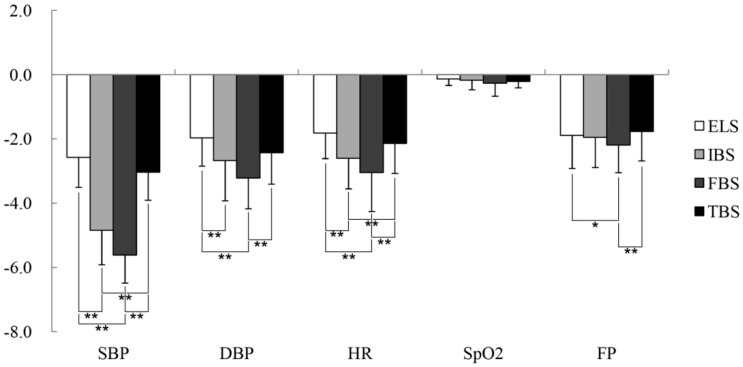
Comparison of physiological index changes among the experimental groups. ELS: exhibition leaf stage, IBS: initial bloom stage, FBS: full bloom stage, and TBS: terminal bloom stage. SBP: systolic blood pressure, DBP: diastolic blood pressure, HR: heart rate, SpO_2_: blood oxygen saturation, and FP: fingertip pulse. N = 74, mean ± SD. * *p* < 0.05, ** *p* < 0.01, one-way ANOVA.

Results of the paired samples *t*-test indicated that there were significant differences in the systolic blood pressure (SBP) (*p* < 0.01 for all stages, with Cohen’s *d* varying from 0.21 to 0.45), diastolic blood pressure (DBP) (*p* < 0.01 for all stages, *d* = 0.24–0.51), heart rate (HR) (*p* < 0.05 for all stages, *d* = 0.20–0.37), and fingertip pulse (FP) (*p* < 0.05 for all stages, *d* = 0.18–0.28) of middle-aged and elderly people in the test groups relative to the control groups in the four periods. However, the SpO_2_ (*p* > 0.05) showed no significant differences (Figure 4, Table 2). Compared with the other three periods, the changes in the SBP (*p* = 0.00, *d* = 0.45), DBP (*p* = 0.00, *d* = 0.51), HR (*p* = 0.00, *d* = 0.37), and FP (*p* = 0.00, *d* = 0.28) were the largest for the 15-minute peony-viewing program at the FBS. One-way analysis of variance revealed that viewing the tree peonies decreased SBP (*p* = 0.00, *η_p_*^2^ = 0.64), DBP (*p* = 0.00, *η_p_*^2^ = 0.16), HR (*p* = 0.00, *η_p_*^2^ = 0.18), and FP (*p* = 0.01, *η_p_*^2^ = 0.02) at different phenological stages. Furthermore, there were no significant alterations in the SBP, DBP, HR, and FP between the IBS and the FBS. The changes in DBP and FP between the initial bloom stage (IBS) and the terminal bloom stage (TBS) were not significant after participants viewed tree peonies, and the changes of FP caused by initial bloom stage (IBS) and exhibition leaf stage (ELS) were not significant (Figure 5). 

**Figure 6:**
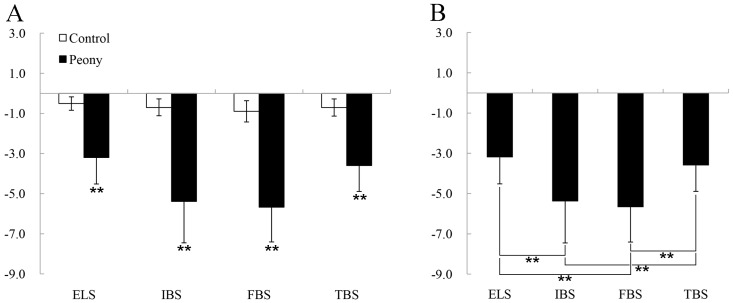
STAI-S scores between the experimental (peony) and the control groups in the ELS (exhibition leaf stage), IBS (initial bloom stage), FBS (full bloom stage), and TBS (terminal bloom stage). (**A**) Changes of pre- and post-viewing measurement. N = 74, mean ± SD. ** *p* < 0.01, paired-sample *t*-test. (**B**) comparison of STAI-S scores among the experimental groups. N = 74, mean ± SD. ** *p* < 0.01, one-way ANOVA.

**Figure 7:**
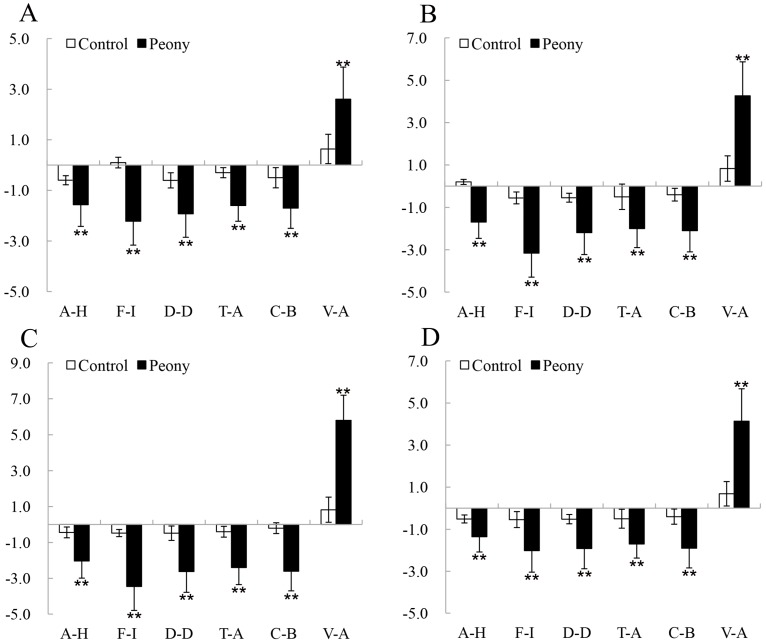
The pre- and post-viewing changes of POMS scores during the experimental (peony) and control groups in the (**A**) ELS (exhibition leaf stage), (**B**) IBS (initial bloom stage), (**C**) FBS (full bloom stage), and (**D**) TBS (terminal bloom stage). A–H: anger–hostility, F–I: fatigue–inertia, D–D: depression–dejection, T–A: tension–anxiety, C–B: confusion–bewilderment, and V–A: vigor–activity. N = 74, mean ± SD. ** *p* < 0.01, paired-sample *t*-test.

**Figure 8:**
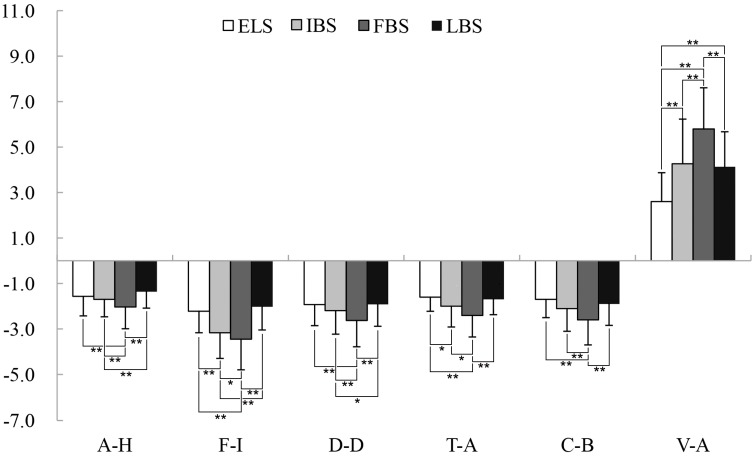
Comparison of POMS scores among the experimental groups. ELS: exhibition leaf stage, IBS: initial bloom stage, FBS: full bloom stage, and TBS: terminal bloom stage. A–H: anger–hostility, F–I: fatigue–inertia, D–D: depression–dejection, T–A: tension–anxiety, C–B: confusion–bewilderment, and V–A: vigor–activity. N = 74, mean ± SD. * *p* < 0.05, ** *p* < 0.01, one-way ANOVA.

Paired *t*-tests showed that all of the POMS and STAI scores revealed significant changes after viewing tree peonies in the different periods. Compared with the control groups, three of the negative subscales of the POMS, anger–hostility (A–H) (*p* < 0.01 for all stages, *d* = 0.67–1.19), fatigue–inertia (F–I) (*p* < 0.01 for all stages, *d* = 0.81–1.77), depression–dejection (D–D) (*p* < 0.01 for all stages, *d* = 0.64–1.24), tension–anxiety (T–A) (*p* < 0.01 for all stages, *d* = 0.87–1.13), and confusion–bewilderment (C–B) (*p* < 0.01 for all stages, *d* = 0.81–1.07), substantially decreased from the pretest to posttest. Conversely, the positive mood state (vigor–activity (V–A)) significantly increased (*p* < 0.01 for all stages, *d* = 0.52–1.80), and the state anxiety subscale of the STAI measurement exhibited improvement (*p* < 0.01 for all stages, *d* = 0.38–1.22). Meanwhile, the changes of psychological indices, as described above, were the greatest values in the FBS (Figure 6A, Figure 7, Table 3). The changes of the POMS and STAI scores were significant (*p* < 0.01) among the experimental groups. One-way analysis of variance revealed that viewing the tree peonies in the four periods reduced anxiety (*p* = 0.00, *η_p_*^2^ = 0.29), A–H (*p* = 0.00, *η_p_*^2^ = 0.24), F–I (*p* = 0.00, *η_p_*^2^ = 0.24), D–D (*p* = 0.04, *η_p_*^2^ = 0.06), T–A (*p* = 0.00, *η_p_*^2^ = 0.06), and C–B (*p* = 0.00, *η_p_*^2^ = 0.07), and it beneficially affected V–A (*p* = 0.00, *η_p_*^2^ = 0.35). In addition, there were no significant differences in the anxiety of STAI scores between IBS and FBS. The changes between initial bloom stage (IBS) and exhibition leaf stage (ELS) in A–H, D–D, and C–B of POMS scores were not significant after participants viewed tree peonies, and the changes of T–A, C–B, and V–A caused by initial bloom stage (IBS) and terminal bloom stage (TBS) were not significant (Figure 6B and Figure 8). 

## 4. Discussion

This study explored the physiological and psychological responses of middle-aged and elderly individuals to tree peonies. Compared to the measurements for the control groups, physiological parameters (SBP, DBP, HR, and FP) and POMS (A–H, D–D, T–A, C–B, F–I, and V–A) and STAI scores were determined to have changed significantly after the peony-viewing program in each period, especially in the FBS. These outcomes, showing that visual interaction with nature or plants could relax people and reduce stress, were similar to those found in previous studies [11,12,13,14,15,16,17,18,24,25,26,34,36,47]. However, our results suggest that the differences in SpO_2_ levels were not significant after viewing tree peonies. SpO_2_ is a relative measurement index of the amount of oxygen dissolved or carried in the blood. It indicates whether a person has a sufficient supply of oxygen and reflects the level of cardiorespiratory health [45]. SpO_2_ can be determined by a pulse oximeter, which is often attached to the finger for measurement purposes. It remains unclear why SpO_2_ did not change significantly in our study. It might be simply that the peony-viewing activity had limited effects on SpO_2_. Alternatively, this research may be unlike previous studies in which SpO_2_ was evaluated under different medical situations (anesthesia, sleep apnea, and parturition) [48] in contrast to the green garden environment in this study.

It is worth mentioning that our research, differing from the previous studies, has been completed in different phenological periods of the tree peonies. With the opening of peony flowers, the morphology and aroma concentration of peony flowers are constantly changing. From the perspective of plant landscape, we consider that the present study suggested two key findings. First, the changes of psycho-physiological parameters were greatest at the full blooming stage. This indicated that rich landscape structures (complex flower types, big flowers, and various colors) could provide a more relaxed environment. Second, the aroma released from the peony flowers may form a smellscape [49]. The peony smellscape may lead to people’s emotional relaxation, as previously described [50]. Therefore, we will explore the influence of peony flower types, colors, and volatile organic compounds on human health in future studies. The information can greatly contribute to the development of color therapy and aromatherapy with tree peonies. On this basis, we suggest that landscape architects properly and reasonably increase the application of tree peonies in the designs of landscape (e.g., the designs of parterres and flower borders) to create an aesthetic and health-promoting environment. 

The present findings provided scientific evidence on the positive psycho-physiological benefits of peony-viewing to middle-aged and elderly individuals by investigating a small sample size in a campus environment, not a large sample size in a natural environment [16,17,26]. However, the tree peony flowering period lasts usually only 15 to 20 days. Thus, people cannot view peony flowers all year round. We should enrich the scenery of the peony garden by planting fall foliage plants to make up for the depressed landscape after the peony flowers fade. Because the great effects revealed in this study may be due to the compositions of different colors, fall foliage may have similar effects due to the contrast of colors, which may need a further study. We also request that the university consider increasing the availability of peony gardening operation activities in the peony garden, such as planting, weeding, watering, fertilizing, pruning, and harvesting. Peony-based activities provide opportunities for social interaction between middle-aged and elderly people, further contributing to their mental health improvement. In addition, we consider that the horticulturist can cultivate the tree peonies by forcing or retarding culture and make peony flowers bloom in all seasons besides the spring. 

We must admit that the present study does have a few limitations, however. First, each participant visited the tree peonies only once for a short time, so that the differences of the data at different times (morning or afternoon) of day and the long-term effects on participants still remain unclear. Second, the lack of participants’ personal information of confounding variables such as socioeconomic status, cultural background (e.g., peony culture researcher), group characteristic (e.g., hypertension, diabetes, heart disease, etc.), and personality (e.g., peony lover) potentially affects the accuracy and reliability of the experimental results. Additionally, the present study only used blood pressure, heart rate, blood oxygen saturation, and fingertip pulse to demonstrate the internal state of participants. Future studies should include the assessment of other physiological indices, such as brain activity, eye tracking, electrical conductivity of the skin, and salivary cortisol.

## 5. Conclusions

Our study revealed that the short peony-viewing program elicited a significant change in physiological and psychological indicators of middle-aged and elderly individuals among various experimental groups. However, no significant changes in SpO_2_ occurred. Simultaneously, we found that the promotional effects of the peony-viewing program on the subjects’ physical and mental health, in sequence, were FBS > IBS > ELS and TBS. We thus concluded that middle-aged and elderly people could substantially benefit from short peony-viewing programs in terms of body and mind.

## Figures and Tables

**Table 1:** Summary of sample characteristics.

Variable	Group	Number of Participants	Percentage
**Gender**	Male	37	50.00%
	Female	37	50.00%
**Ages**	40–60 years old	40	54.05%
	60–80 years old	34	45.95%

**Table 2:** The pre- and post-measurement variable values of participants’ physiological indices in the control and experimental groups across four stages.

Physiological Indices	Stage	Control Group				Experimental Group			
		Pretest	Posttest	*p*	Effect Size	Pretest	Posttest	*p*	Effect Size
**SBP**	**ELS**	125.9 ± 10.2	125.2 ± 9.4	0.190	0.07	127.6 ± 11.4	125.2 ± 11.3	0.000 **	0.21
	**IBS**	123.8 ± 11.3	122.9 ± 10.2	0.110	0.08	126.7 ± 10.4	121.9 ± 10.4	0.000 **	0.45
	**FBS**	125.1 ± 11.1	124.1 ± 9.9	0.130	0.10	128.1 ± 12.2	122.5 ± 12.3	0.000 **	0.45
	**TBS**	124.3 ± 9.9	123.5 ± 9.5	0.127	0.08	126.8 ± 10.6	123.8 ± 10.5	0.000 **	0.28
**DBP**	**ELS**	72.1 ± 7.8	71.5 ± 7.6	0.097	0.07	78.6 ± 8.1	76.6 ± 8.0	0.000 **	0.24
	**IBS**	76.6 ± 9.2	76.1 ± 9.1	0.529	0.05	77.2 ± 6.6	74.5 ± 6.5	0.000 **	0.41
	**FBS**	72.6 ± 10.6	71.5 ± 10.0	0.094	0.11	76.8 ± 6.4	73.6 ± 6.3	0.000 **	0.51
	**TBS**	74.7 ± 7.3	73.9 ± 7.2	0.053	0.12	77.5 ± 7.5	75.0 ± 7.4	0.000 **	0.34
**HR**	**ELS**	79.4 ± 9.9	78.7 ± 9.9	0.084	0.07	78.4 ± 9.1	76.6 ± 9.1	0.000 **	0.20
	**IBS**	78.9 ± 8.9	78.1 ± 9.5	0.088	0.08	78.9 ± 8.7	76.3 ± 8.6	0.000 **	0.30
	**FBS**	72.4 ± 5.6	71.5 ± 6.7	0.152	0.14	78.1 ± 7.9	75.1 ± 7.9	0.000 **	0.37
	**TBS**	80.2 ± 11.1	79.5 ± 11.3	0.176	0.06	77.3 ± 10.0	75.2 ± 9.9	0.000 **	0.21
**SpO_2_**	**ELS**	97.9 ± 1.1	97.8 ± 1.1	0.595	0.09	97.9 ± 1.2	97.7 ± 1.4	0.465	0.15
	**IBS**	97.9 ± 1.1	97.7 ± 1.2	0.344	0.17	97.1 ± 1.4	96.9 ± 1.3	0.280	0.14
	**FBS**	97.5 ± 1.3	97.3 ± 1.3	0.096	0.15	97.9 ± 1.0	97.6 ± 1.1	0.068	0.28
	**TBS**	98.1 ± 1.3	97.9 ± 1.2	0.176	0.16	97.8 ± 1.3	97.7 ± 1.3	0.364	0.08
**FP**	**ELS**	72.0 ± 6.2	71.2 ± 7.3	0.172	0.12	78.2 ± 10.9	76.2 ± 10.8	0.000 **	0.18
	**IBS**	77.4 ± 8.9	76.7 ± 8.4	0.105	0.08	79.9 ± 10.1	77.9 ± 9.9	0.000 **	0.20
	**FBS**	79.1 ± 9.6	78.4 ± 9.6	0.123	0.07	78.3 ± 7.7	76.1 ± 7.9	0.000 **	0.28
	**TBS**	77.1 ± 11.2	76.4 ± 11.8	0.064	0.06	77.7 ± 9.2	75.9 ± 9.1	0.000 **	0.19

N = 74, mean ± SD, ** *p* < 0.01, paired-sample *t*-test. ELS: exhibition leaf stage, IBS: initial bloom stage, FBS: full bloom stage, and TBS: terminal bloom stage. SBP: systolic blood pressure, DBP: diastolic blood pressure, HR: heart rate, SpO_2_: blood oxygen saturation, and FP: fingertip pulse.

**Table 3:** The pre- and post-measurement variable values of participants’ psychological indices in the control and experimental groups across four stages.

Psychological Indices	Stage	Control Group				Experimental Group			
		Pretest	Posttest	*p*	Effect Size	Pretest	Posttest	*p*	Effect Size
**A–H**	**ELS**	8.4 ± 2.4	8.0 ± 2.3	0.059	0.17	8.6 ± 1.8	7.1 ± 1.5	0.000 **	0.91
	**IBS**	7.9 ± 2.2	8.1 ± 2.5	0.438	0.08	8.6 ± 1.5	6.9 ± 1.3	0.000 **	1.19
	**FBS**	8.4 ± 2.6	8.1 ± 2.1	0.185	0.13	8.9 ± 2.0	6.9 ± 1.7	0.000 **	1.07
	**TBS**	8.4 ± 2.6	8.0 ± 2.3	0.084	0.16	8.6 ± 2.3	7.1 ± 1.9	0.000 **	0.67
**F–I**	**ELS**	8.3 ± 2.7	8.4 ± 2.7	0.838	0.04	8.8 ± 2.4	6.6 ± 2.0	0.000 **	1.00
	**IBS**	8.2 ± 2.5	7.7 ± 2.5	0.087	0.20	9.6 ± 2.0	6.4 ± 1.6	0.000 **	1.77
	**FBS**	8.6 ± 2.7	8.1 ± 2.5	0.051	0.19	10.1 ± 2.8	6.6 ± 2.3	0.000 **	1.37
	**TBS**	8.4 ± 2.7	7.9 ± 2.6	0.063	0.19	8.8 ± 2.7	6.8 ± 2.3	0.000 **	0.81
**D–D**	**ELS**	9.1 ± 3.0	8.7 ± 3.2	0.084	0.13	8.6 ± 2.0	6.7 ± 1.5	0.000 **	1.07
	**IBS**	8.9 ± 3.1	8.5 ± 2.8	0.075	0.14	9.6 ± 3.0	7.4 ± 2.4	0.000 **	0.81
	**FBS**	8.7 ± 3.3	8.3 ± 3.0	0.091	0.13	9.6 ± 2.6	7.0 ± 2.0	0.000 **	1.24
	**TBS**	8.6 ± 2.9	8.3 ± 3.0	0.059	0.10	9.5 ± 3.1	7.6 ± 2.7	0.000 **	0.64
**T–A**	**ELS**	7.9 ± 2.1	7.6 ± 1.9	0.082	0.15	8.6 ± 1.7	7.1 ± 1.4	0.000 **	0.96
	**IBS**	8.0 ± 2.5	7.6 ± 2.0	0.060	0.18	8.9 ± 2.0	6.9 ± 1.6	0.000 **	1.10
	**FBS**	8.1 ± 2.6	7.7 ± 2.5	0.063	0.16	9.3 ± 2.4	6.9 ± 1.8	0.000 **	1.13
	**TBS**	8.6 ± 2.7	8.0 ± 2.6	0.065	0.23	8.7 ± 2.0	7.0 ± 1.9	0.000 **	0.87
**C–B**	**ELS**	8.1 ± 2.8	7.6 ± 2.6	0.078	0.19	8.9 ± 1.9	7.1 ± 1.8	0.000 **	0.97
	**IBS**	8.3 ± 2.8	8.0 ± 2.3	0.070	0.12	9.6 ± 3.1	7.5 ± 2.6	0.000 **	0.73
	**FBS**	8.6 ± 3.1	8.4 ± 3.1	0.260	0.06	9.4 ± 2.7	6.8 ± 2.1	0.000 **	1.07
	**TBS**	8.4 ± 2.7	8.0 ± 2.4	0.155	0.16	9.2 ± 2.5	7.3 ± 2.2	0.000 **	0.81
**V–A**	**ELS**	19.1 ± 5.2	19.7 ± 4.4	0.099	0.12	18.0 ± 4.9	20.6 ± 4.8	0.000 **	0.52
	**IBS**	19.7 ± 5.1	20.4 ± 4.5	0.091	0.15	18.3 ± 4.2	22.6 ± 4.7	0.000 **	0.96
	**FBS**	18.1 ± 4.4	18.9 ± 4.4	0.054	0.18	17.6 ± 3.0	23.4 ± 3.4	0.000 **	1.80
	**TBS**	19.2 ± 5.3	19.8 ± 4.6	0.072	0.12	17.5 ± 4.3	21.6 ± 4.5	0.000 **	0.93
**Anxiety (STAI-S)**	**ELS**	36.5 ± 6.2	35.9 ± 6.5	0.061	0.09	37.9 ± 8.3	34.8 ± 8.4	0.000 **	0.38
	**IBS**	35.9 ± 8.4	35.2 ± 7.7	0.064	0.10	38.1 ± 7.1	32.6 ± 6.7	0.000 **	0.83
	**FBS**	37.1 ± 6.2	36.4 ± 6.4	0.066	0.11	37.0 ± 4.7	31.4 ± 4.6	0.000 **	1.22
	**TBS**	35.6 ± 6.3	35.0 ± 6.7	0.052	0.09	38.2 ± 6.5	34.6 ± 6.5	0.000 **	0.55

N = 74, mean ± SD, ** *p* < 0.01, paired-sample t-test. ELS: exhibition leaf stage, IBS: initial bloom stage, FBS: full bloom stage, and TBS: terminal bloom stage. A–H: anger–hostility, F–I: fatigue–inertia, D–D: depression–dejection, T–A: tension–anxiety, C–B: confusion–bewilderment, and V–A: vigor–activity.
